# Design, Immune Responses and Anti-Tumor Potential of an HPV16 E6E7 Multi-Epitope Vaccine

**DOI:** 10.1371/journal.pone.0138686

**Published:** 2015-09-21

**Authors:** Liliane Maria Fernandes de Oliveira, Mirian Galliote Morale, Agatha A. Muniz Chaves, Aline Marques Cavalher, Aline Soriano Lopes, Mariana de Oliveira Diniz, Alessandra Soares Schanoski, Robson Lopes de Melo, Luís Carlos de Souza Ferreira, Maria Leonor S. de Oliveira, Marilene Demasi, Paulo Lee Ho

**Affiliations:** 1 Laboratório de Biotecnologia Molecular I, Instituto Butantan, Av. Vital Brasil 1500, São Paulo-SP, Brazil; 2 Laboratório Especial de Toxinologia Aplicada-CeTICS, Instituto Butantan, Av. Vital Brasil 1500, São Paulo-SP, Brazil; 3 Departamento de Microbiologia, Instituto de Ciências Biomédicas, Universidade de São Paulo, São Paulo-SP, Brazil; 4 Laboratório de Bioquímica e Biofísica, Instituto Butantan, Av. Vital Brasil 1500, São Paulo-SP, Brazil; Istituto Superiore di Sanita, ITALY

## Abstract

Cervical cancer is a common type of cancer among women worldwide and infection with high-risk human papillomavirus (HPVs) types represents the major risk factor for the etiopathogenesis of the disease. HPV-16 is the most frequently identified HPV type in cervical lesions and expression of E6 and E7 oncoproteins is required for the uncontrolled cellular proliferation. In the present study we report the design and experimental testing of a recombinant multi-epitope protein containing immunogenic epitopes of HPV-16 E6 and E7. Tumor preventive assays, based on the engraftment of TC-1 cells in mice, showed that the E6E7 multi-epitope protein induced a full preventive anti-tumor protection in wild-type mice, as well as in mice deficient in expression of CD4^+^ T cells and TLR4 receptor. Nonetheless, no anti-tumor protection was observed in mice deficient in CD8^+^ T cells. Also, the vaccine promoted high activation of E6/E7-specific T cells and in a therapeutic-approach, E6E7 protein conferred full anti-tumor protection in mice. These results show a potential use of this E6E7 multi-epitope antigen as a new and promising antigen for the development of a therapeutic vaccine against tumors induced by HPV.

## Introduction

Cervical cancer is the fourth most common type of cancer among women worldwide [1]. Persistent infection with high-risk human papillomavirus (HPVs) is the primary risk factor for the development of the disease. As an attempt to reduce the high incidence of this infection and the deaths resulting from cervical cancer, as well as other cancer types induced by HPV, two prophylactic vaccines based on L1 antigen have been approved in more than 100 countries. Although both vaccines have proven to be highly effective in preventing HPV infection, neither one shows therapeutic effects to established HPV infections. Indeed, thousands of people still die each year with the disease despite advances in surgery, radiotherapy or chemotherapy treatments [2,3].

An alternative approach to treat lesions associated with HPV infection is based on therapeutic vaccines to tumors induced by HPV. Unlike anti-virus prophylactic vaccines based on the induction of neutralizing antibodies, therapeutic anti-tumor vaccines require induction of cell-mediated immune responses capable to identify and eliminate abnormal cells. To achieve an effective treatment, the therapeutic vaccines depend on a close cooperation between the innate and adaptive immune system, in particular, antigen-presenting cells (APC), CD4^+^ helper T cells and CD8^+^ cytotoxic T lymphocytes (CTLs) [4].

E6 and E7oncoproteins are the targets for immunotherapy against tumors induced by papillomavirus. These proteins are exclusively expressed by the infected tumor cells and represent ideal targets for cytotoxic responses induced by vaccinated subjects.

To increase the magnitude and quality of E6 and E7-specific immune response, we developed a therapeutic vaccine candidate employing a recombinant protein consisting of a string of multi immunogenic T cell epitopes of E6 and E7 (E6E7 vaccine). The results indicate that this approach may contribute to the development of vaccines targeting tumors induced by papillomavirus.

## Materials and Methods

### Mice

Female, 6–10 weeks old, wild-type, CD4^+^ or CD8^+^ T-cells knockout C57BL/6 mice and C57BL10/ScCr (TLR4 knockout) mice were purchased from Institute of Biomedical Science IV-USP, São Paulo, and kept in pathogen-free condition in the animal facility of Butantan Institute. These experiments were conducted according to the Ethical Principles of Animal Experimentation adopted by Brazilian College of Animal Experimentation (COBEA) and was approved by Animal Ethic Committee Guidelines of Butantan Institute (protocol number: 649/09).

### Cell lines

TC-1 cells, generated by co-transformation of C57BL/6 mouse lung epithelial cells with HPV16 E6 and E7 and an activated ras oncogene were kindly provided by T. C. Wu, Johns Hopkins University, Baltimore, MD. The TC-1 cell line was propagated as previously described [5]. Before inoculation, TC-1 cells were harvested by trypsinization, washed three times with PBS pH 7.5 buffer and suspended in serum free DMEM media (Gibco) at a concentration of 7.5 x 10^5^ cells/mL.

### Peptides and antibodies

E6 and E7 synthetic peptides representing H2-D^b^ and H2-K^b^ restricted CD8 T-cells epitopes [E6 (VYDFAFRDL^49-57^; DKKQRFHNI^127-135^) and E7 (RAHYNIVTF^49-57^; LCVQSTHVD^67-75^)] of HPV16 were synthesized at the Center for Applied Toxinology–CAT, Butantan Institute as described by Carpino and Han [6]. For the Elispot analysis, mouse IFN-γ ELISPOT kits (BD Bioscience) were used.

### Plasmid construction

For construction of a vector expressing the E6E7 multi-epitope protein, the synthetic coding sequence of E6E7Ub protein was excised from pUC57 (GenScript Inc.) using *BamHI*/*PvuII* and cloned into pAE vector [7] at the same sites. The pAE-E6E7 plasmid was constructed by excising of ubiquitin (Ub) sequence from pAE-E6E7Ub through digestion with *EcoRI* enzyme designed to flank the ubiquitin cDNA. The construction was designed to express the recombinant protein with a 6 x His tag at the N-terminus. The nucleotides sequence was confirmed by DNA sequencing using Big Dye Terminator Reaction Kit version 3.1 and ABI PRISM 3100 Genetic Analyzer (Applied Biosystems).

### Expression, purification and characterization of recombinant proteins

The E6E7 recombinant protein was expressed in the BL21(DE3) Star pLysS (Novagen) *E*. *coli* strain. Isolated colonies grown on LB agar were transferred into 0.5 L of LB with ampicillin and incubated in a shaker at 37°C until the optical density at 600 nm (OD_600_) reached 0.6. Protein expression was induced by adding 1 mM isopropyl-β-thiogalactopyranoside and the cultures were incubated at 37°C for 4 h. Cells were harvested by centrifugation and lysed using a French press. Bacterial inclusion bodies were washed in washing buffer (PBS pH 7.5, 250 mM NaCl and 2 M urea) and solubilized with binding buffer (PBS pH 7.5, 250 mM NaCl, 8 M urea, 5 mM β-mercaptoethanol and 5 mM imidazole).

The recombinant protein was purified on Chelating-Sepharose^™^ Fast Flow (GE Healthcare). The columns with adsorbed protein were washed three times with binding buffer containing 60 mM, 80 mM and 100 mM imidazole. The E6E7 protein was eluted with elution buffer (PBS pH 7.5, 250 mM imidazole and 8 M urea). Stepwise dialysis was performed until urea reached a concentration of 2 M. The protein was quantified by spectrophotometry (NanoDrop, Thermo Scientific Inc.) according to its molar extinction coefficient.

The recombinant protein was characterized by SDS-PAGE and mass spectrometry (S1 Material and Methods).

### Prophylactic immunization

Groups of five C57BL/6 mice were immunized subcutaneously (s.c) in the right flank with three doses of E6E7 (18 μg/dose) or 100 μl PBS/2 M urea at 14 days intervals. After two weeks from the last dose, mice were challenged (s.c) with 7.5 x 10^4^ TC-1 cells into the right flank. Tumor growth was monitored daily. The mice were euthanized by CO_2_ inhalation once the tumors reached 1.5–2.0 cm in size. TLR4 (5–6 mice/group), CD4^+^ T-cells (8 mice/group) or CD8^+^ T-cells knockout (5 mice/group) animals were immunized with E6E7 or PBS/2 M urea, following the above protocol. A group of five wild-type C57BL/6 mice immunized with PBS/2 M urea was used as a control of tumor growth in all these cases.

### Therapeutic immunization

Groups of five C57BL/6 animals were first injected (s.c) into the right flank with 7.5 x 10^4^ TC-1 cells. After three days, the immunization protocol was initiated, being injected (s.c) three doses of E6E7 (18 μg/dose) or 100 μL PBS/2 M urea at 7 days intervals. Tumor growth was monitored daily using a caliper, measuring two opposing diameters. The results are presented in measurement as mm^2^ (length x width). Following the above protocol of therapeutic immunization, TLR4 knockout animals were immunized with E6E7 (4 mice) or PBS/2 M urea (4 mice). In parallel, a group of five wild-type C57BL/6 mice immunized with PBS/2 M urea was used as a control of tumor growth.

### Evaluation of cellular immune response

Immune response generated by E6E7 vaccines was evaluated using enzyme-linked immunosorbent spot (ELISPOT) assays to detect cells secreting IFN-γ.

The ELISPOT assay was performed using peripheral blood mononuclear cells (PBMCs) from animals immunized according to prophylactic protocol and challenged with TC-1 cells 20 days before the bleeding. Blood was treated with the ACK lysing buffer (0.15 M NH_4_Cl, 10 mM KHCO_3_ and 0.1 mM EDTA, pH 7.2), centrifuged and suspended in serum free RPMI media (Gibco). 10^5^ cells were plated in a 96-well plate (Corning Life Science) previously coated with anti-mouse IFN-γ (BD Bioscience) and incubated for 48 hours at 37°C in 200 μl of RPMI supplemented with 2% fetal bovine serum, 10 ng/mL IL-2 and 10^−6^ M β-mercaptoethanol. E6-specific (VYDFAFRDL^49-57^; DKKQRFHNI^127-135^) and/or E7-specific (RAHYNIVTF^49-57^; LCVQSTHVD^67-75^) peptides were then used as stimuli at a concentration of 2μg/mL for 48 hours. After incubation, the spots were revealed according to the manufacturer instructions (mouse IFN-γ ELISPOT kits—BD Bioscience). Plates were dried and the number of spots counted.

### Statistical analyses

Statistical significance was performed using Student's t test (Elispot assay) or Mantel-Cox curves using log-rank tests (Percentage of tumor-free mice). P < 0.05 was considered significant. All analysis and graphics were done using GraphPad Prism version 5.

## Results

### Design and production of E6E7 multi-epitope protein

**Fig 1:**
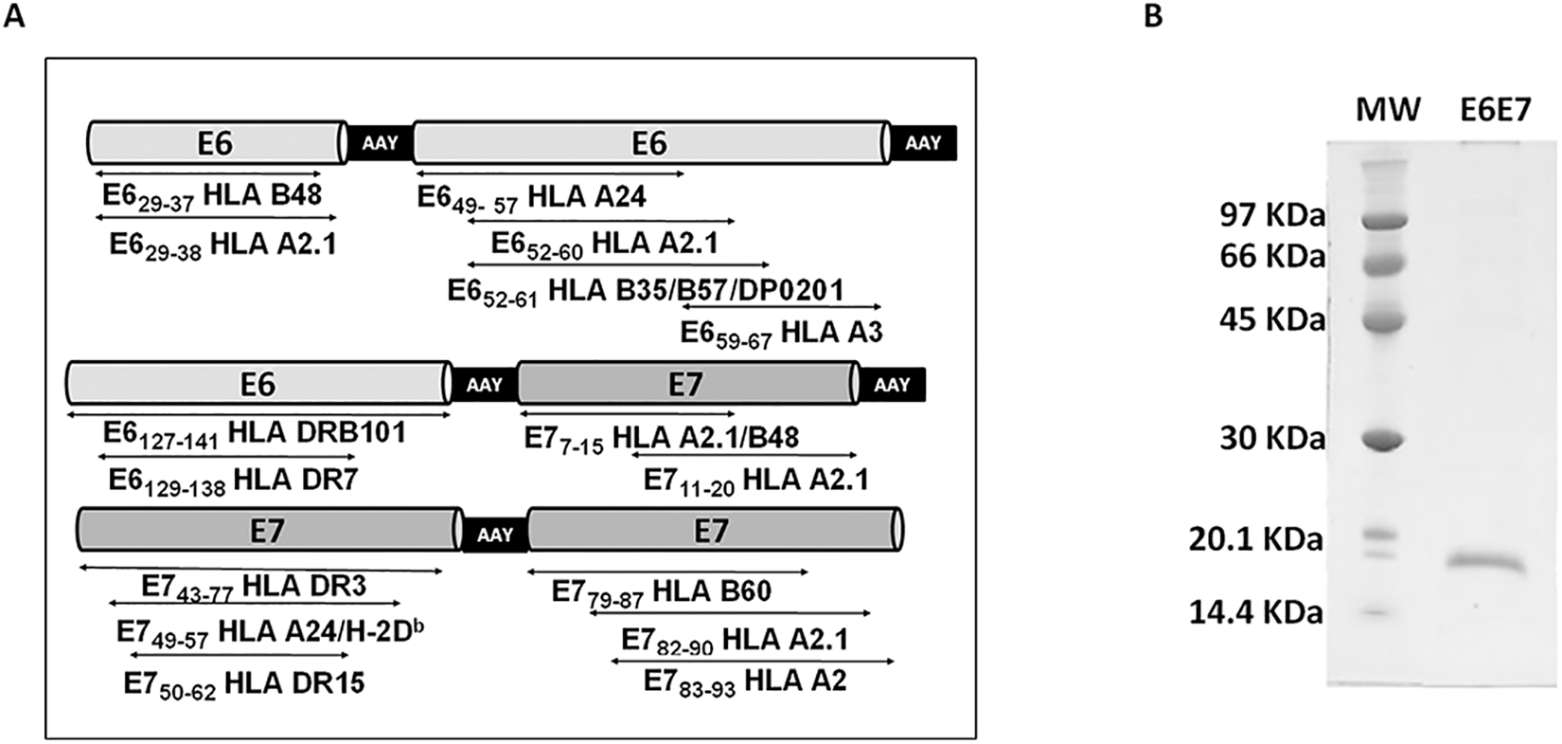
Design and purification of the vaccine antigen. (A) Scheme of the designed E6E7 antigen. Ala-Ala-Tyr (AAY) spacers were inserted between the epitopes. (B) SDS-PAGE of purified antigen stained with Coomassie Brilliant Blue. MW: molecular mass marker.

The E6E7 antigen was designed to contain HPV E6 and E7 immunogenic epitopes based on published data reporting antigen specific cellular immune responses detected in patients and/or C57BL/6 mice. MHC class I-restricted epitopes were included as an attempt to induce T CD8^+^ responses, known to play an important role in tumor clearance. Also, we added to the E6E7 antigen sequence epitopes that are presented to CD4^+^ T-cells by MHC class II known to induce long lasting cytotoxic responses [8–15] (Fig 1A).

Considering the high polymorphism of HLA genes in the human population, we elected E6 and E7 regions that presented the higher number of established epitopes covering several human HLA types [16]. Thereby, the final construct contained the following regions of the HPV16 E6 and E7 proteins: E6 _29–38_, E6_49-67_, E6_127-141_ [9,10,16–21], E7_7-20_, E7_43-77_ and E7_79-93_ [9,11–13,15,19,22] (Table 1). The Ala-Ala-Tyr (AAY) spacers were inserted between the epitope sequences (Fig 1A) to increase proteasome degradation at specific sites, enabling right epitopes to be presented [23].

**Table 1:** E6 and E7 epitopes used to assemble in the E6E7 protein.

Peptide	MHC/HLA	Sequence	Reference
E6_29-37_	B48	TIHDIILEC	[16]
E6_29-38_	A2.1	TIHDIILECV	[16,17]
E6_49-57_	A24	VYDFAFRDL	[18]
E6_52-60_	A2.1	FAFRDLCIV	[9]
E6_52-61_	B35; B57; DP0201	FAFRDLCIVY	[10,19]
E6_59-67_	A3	IVYRDGNPY	[9]
E6_127-141_	DRB101	DKKQRFHNIRGRWTG	[20,21]
E6_129-138_	DR7	KQRFHNIRGR	[10]
E7_7-15_	A2.1; B48	TLHEYMLDL	[9,19]
E7_11-20_	A2.1	YMLDLQPETT	[9,11–13]
E7_43-77_	DR3	GQAEPDRAHYNIVTFCCKCDSTLRLCVQSTHVDIR	[22]
E7_49-57_	A24, H-2Db	RAHYNIVTF	[9, 15]
E7_50-62_	DR15	AHYNIVTFCCKCD	[22]
E7_79-87_	B60	LEDLLMGTL	[19]
E7_82-90_	A2.1	LLMGTLGIV	[9,12]
E7_86-93_	A2	TLGIVCPI	[9,11,12]

The E6E7 antigen with a [His]_6_ tag was expressed as inclusion bodies in *E*.*coli* and purified under denaturing protocol. E6E7 protein showed apparent molecular masses of 17.4 kDa in reducing gel (Fig 1B). The antigen was also characterized by mass spectrometry allowing the identification of E6 and E7 related peptides (S1 Fig and S1 Table).

### Immunoprotective antitumor response

**Fig 2:**
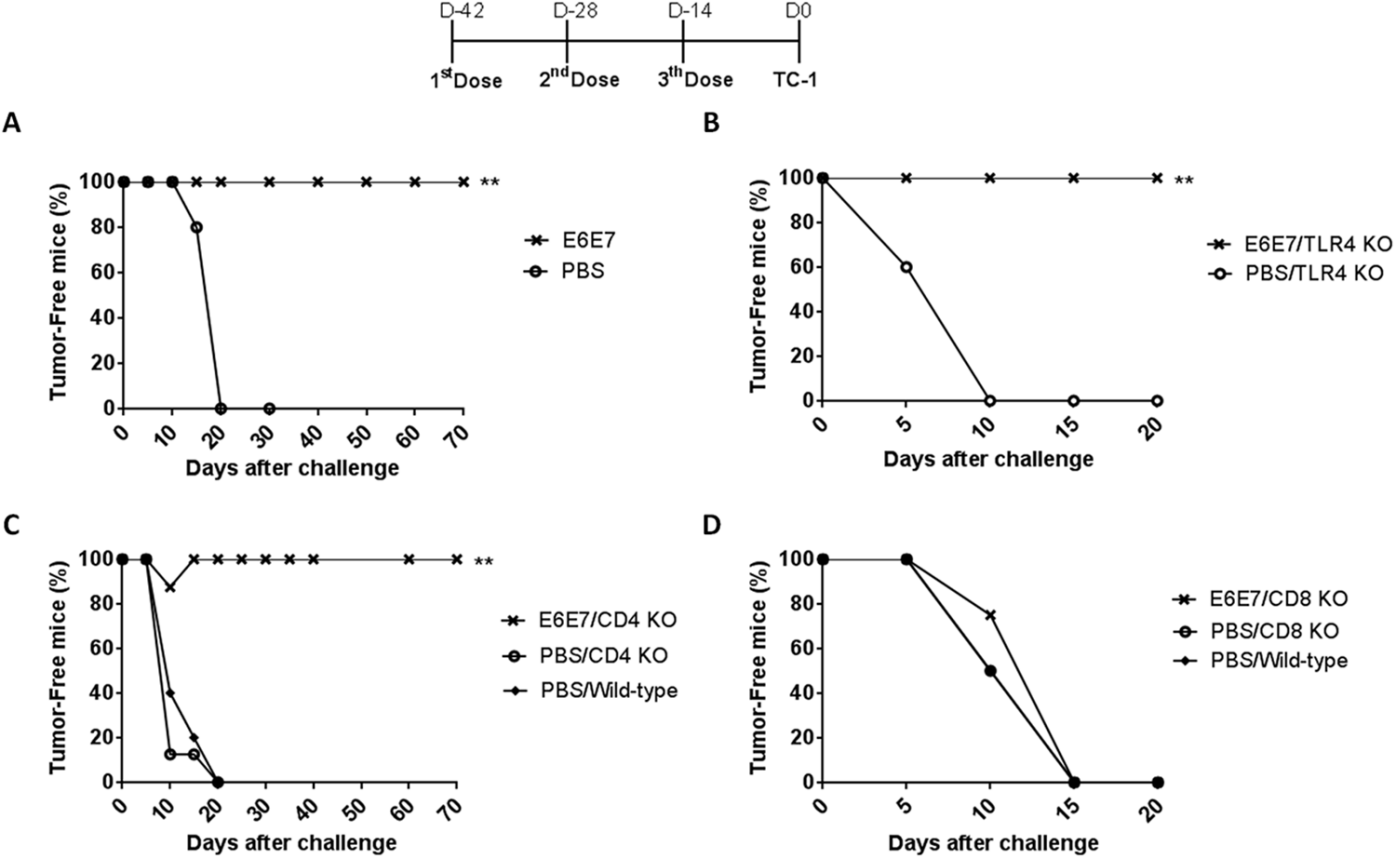
Anti-tumor responses elicited in mice immunized with E6E7 multi-epitope protein. Mice were immunized with three doses of PBS/2M urea (PBS) or E6E7 in intervals of 14 days. Mice were challenged with TC-1 tumor cells two weeks after the last dose. (A) Groups of five C57BL/6 mice were immunized as described. (B) TLR4 KO mice (C57BL10/ScCr) were vaccinated with PBS/2M urea (5 mice, PBS) or E6E7 (6 mice). (C) Groups of eight CD4 KO mice were vaccinated with PBS/2M urea (PBS) or E6E7. (D) Groups of five CD8 KO mice were vaccinated with PBS/2M urea (PBS) or E6E7. In parallel, C57BL/6 wild-type mice (5 mice) were used as a control of tumor growth competence. Asterisk indicates statistically significant percentage of tumor-free animals when compared to control group (** p < 0.005).

The recombinant E6E7 antigen was evaluated for its potential to induce a preventive anti-tumor response in mice engrafted with TC-1 cells. All mice inoculated with PBS/2 M urea developed tumors within fifteen days after challenge. Three doses of E6E7 antigens had the capacity to protect all mice against tumors (Fig 2A).

As the protein antigens were produced in *E*.*coli*, residual lipopolysaccharide (LPS), an agonist of TLR4, are expected to be present in the vaccine preparations. To evaluate the possible impact of LPS contamination on the induced anti-tumor immune responses, we performed prophylactic immunization experiments with TLR4 knockout mice (C57BL10/ScCr). Vaccination with three doses of E6E7 multi-epitope antigen conferred 100% of antitumor protection in TLR4 knockout mice. All mice immunized with PBS/2 M urea developed tumor within 10 days after challenge (Fig 2B). This data showed the antitumor responses induced by the E6E7 multi-epitope vaccine are independent of LPS/TLR4 signaling pathway.

Using CD4^+^ and CD8^+^ T-cells knockout (KO) mice, we investigated the contribution of CD4^+^ and CD8^+^ T-cells to mediate an effective antitumor response in vaccinated animals. Similar to results observed with the wild-type mice (Fig 2A), the protective anti-tumor effects induced by E6E7 vaccination were not affected by the absence of CD4^+^ T-cells (Fig 2C). In contrast, CD8^+^ T-cells KO mice immunized with E6E7or PBS/2 M urea developed tumors within 15 days after challenged (Fig 2D). Thus, CD8^+^ T-cells have a critical role in the protection of mice vaccinated with E6E7 multi-epitope antigens.

### Immunotherapeutic treatment against TC-1 tumor cells

**Fig 3:**
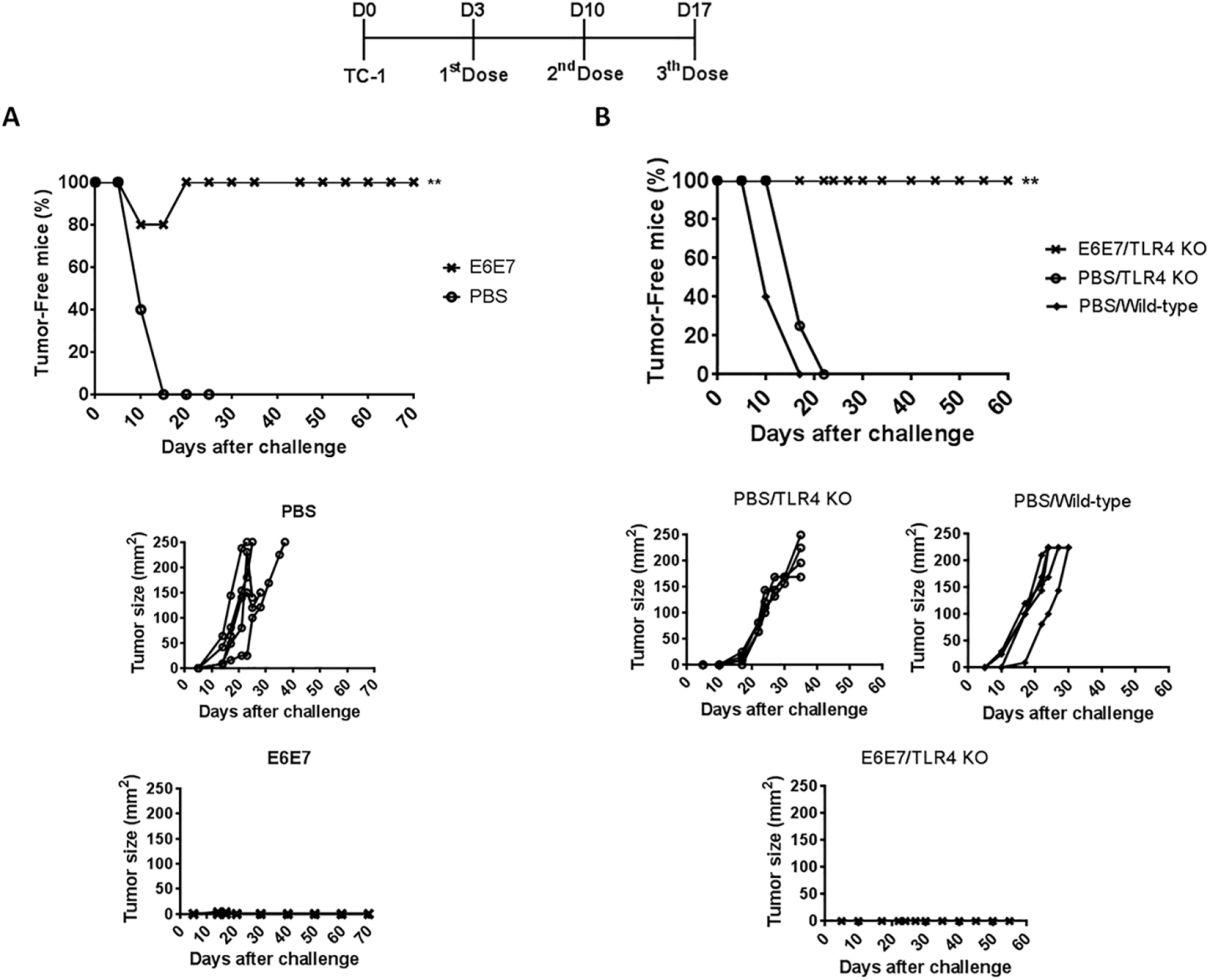
Therapeutic anti-tumor responses elicited in mice immunized with E6E7. (A) Groups of five C57BL/6 mice were treated with three doses of PBS/2M urea (PBS) or E6E7 in intervals of seven days. The first dose was administered three days after the challenge with TC-1 tumor cells. (B) Groups of TLR4 knockout mice were treated with three doses of PBS/2M urea (4 mice, PBS) or E6E7 (4 mice) in intervals of seven days. In parallel, C57BL/6 wild-type mice (5 mice) were used as a control of tumor growth competence. The tumor size in each animal was monitored daily and is given in mm^2^ (length x width).

To evaluate the therapeutic anti-tumor responses induced by the tested vaccine, mice were engrafted with TC-1 cells three days before the first vaccine dose. Under such conditions, the treatment with three doses of E6E7 conferred full anti-tumor protection (Fig 3A). As noted in the graphs of tumor size, all animals treated with PBS/2M urea developed large aggressive tumor within 10–15 days after TC-1 injection (Fig 3A). Likewise, upon challenge, TLR4-deficient and wild-type mice immunized with PBS/2M urea developed palpable tumor within 10–15 days which rapidly reached large size (Fig 3B), whereas the treatment of TLR4-deficient mice with three doses of E6E7 resulted in 100% of therapeutic protection to TC-1 tumors (Fig 3B), showing that the protection does not involve LPS/TLR4 activation.

### Vaccination with E6E7 antigens induces IFN-γ production

**Fig 4:**
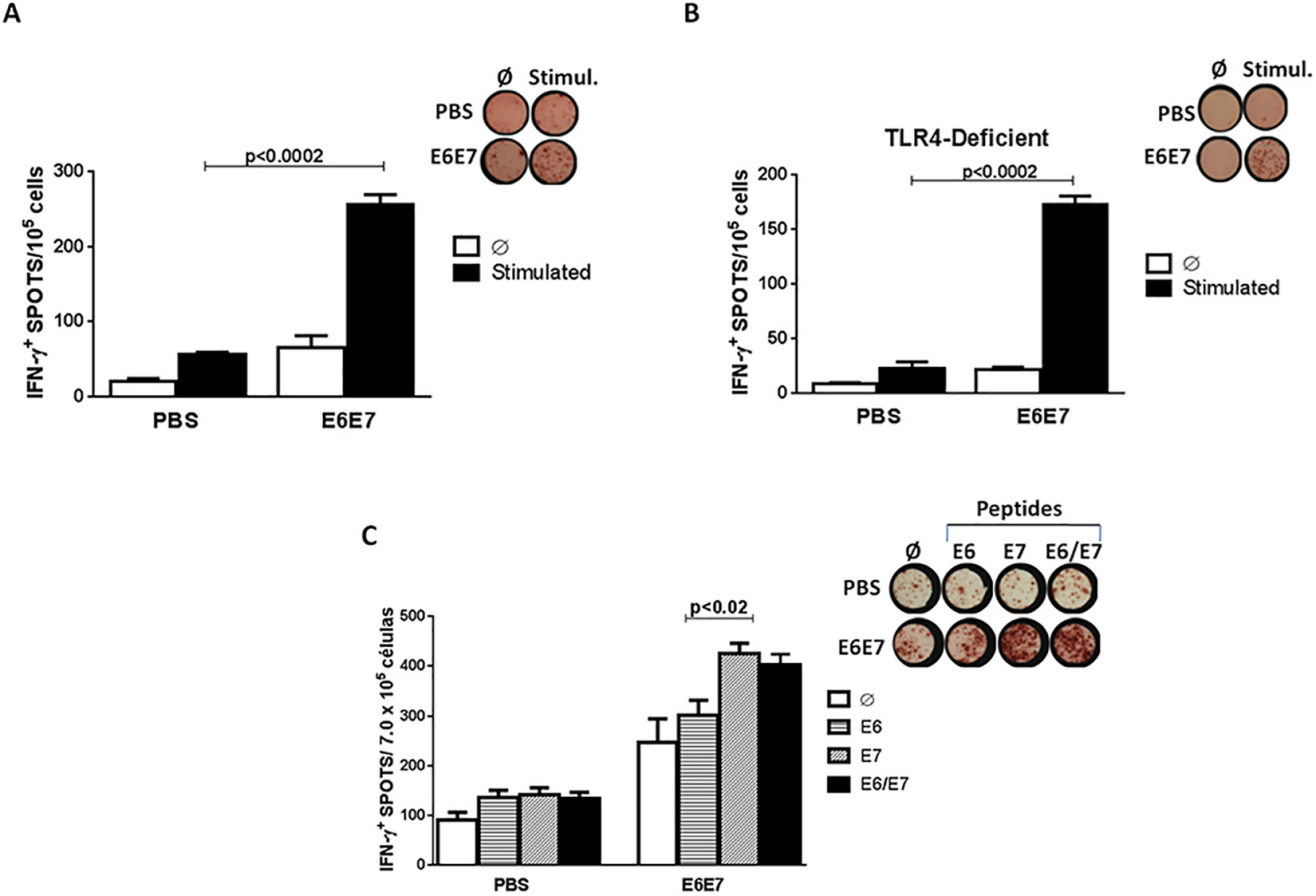
Induction of antigen-specific IFN-γ secretion responses in mice immunized with E6E7. (A) C57BL/6 mice were immunized with three doses of PBS/2M urea (PBS) or E6E7 protein vaccine and challenged with TC-1 tumor cells two w3eeks after the last dose. The ELISPOT assay was performed with PBMC twenty days after the challenge. (B) TLR4 knockout mice were immunized with three doses of PBS/2M urea (PBS) or E6E7 and challenged with TC-1 tumor cells two weeks after the last dose. The ELISPOT assay was performed with PBMC twenty days from the challenge. The cells were stimulated with E6 (VYDFAFRDL^49-57^; DKKQRFHNI^127-135^) and E7 (RAHYNIVTF^49-57^; LCVQSTHVD^67-75^) peptides. (C) Comparative IFN-γ production between PBMC stimulated with E6_49_-_57_ and/or E7_49_-_57_ specific peptides (Stimul.); Ø –Unstimulated cells.

Elispot assays were carried out to detect IFN-γ production by PBMCs cells of mice immunized with E6E7 or PBS/2 M urea and challenged with TC-1 cells. Cells were stimulated *in vitro* with a pool of HPV-16 E6 and E7 peptides. Cells derived from mice immunized with E6E7 secreted higher IFN-γ levels compared to the control group (Fig 4A).

Similar assays were carried out with cells fromTLR4 knockout mice. After stimulation, we observed a marked increase of IFN-γ by PBMCs from animals immunized with E6E7 (Fig 4B), demonstrating that the E6E7 multi-epitope antigen is capable of generating strong IFN-γ production in the absence of LPS/TLR4 signaling pathway.

An Elispot assay was performed to compare the production of IFN-γ between PBMC stimulated with E6 (VYDFAFRDL^49-57^) and/or E7 (RAHYNIVTF^49-57^) specific peptides. We observed high IFN-γ secretion in PBMC cells from mice immunized with the E6E7 vaccine stimulated with E6 peptide or with E7 peptide (Fig 4C). However the IFN-γ production was more pronounced in cells stimulated with the E7-specific peptide, evidencing the immunodominance of this H–2Db-restricted CD8 T cell epitope.

## Discussion

Several efforts have been made to develop vaccines capable of conferring E6/E7 HPV-specific immune responses. Despite some developed vaccines have shown promising results in pre-clinical studies, there is still an urgent need to develop new strategies that can also present clinical efficacy. For that purpose we designed and tested a multi-epitope E6E7 antigen consisting of immunogenic epitopes of HPV-16 E6 and E7 oncoproteins in order to enhance T cells specific immune responses. One advantage of this approach is to cover a large number of immunogenic CTLs epitopes gathered in a single recombinant protein, thereby increasing the possibility of the antigens to be processed and presented by APCs, such as dendritic cells [24]. In addition, we added to the antigen CD4^+^ T helper epitopes to enhance activation of CD8^+^ T-cells and the maintenance of the long term protective immunity [25]. Finally, the design of the antigen included also a variety of promiscuous HLA epitopes enabling a wider response in genetically heterogeneous subjects.

Several peptides and protein-based vaccines containing CTL immunodominant epitopes have been studied and some have been tested in clinical trials. A therapeutic vaccination with thirteen synthetic long peptides (SLPs) containing both CLT and Th cells epitopes of E6 and/or E7 resulted in the induction of robust E7-specific CD8^+^ T and CD4^+^ Th cells responses in animal model [15,26]. In clinical trials, this vaccine admixed with Montanide ISA-51 promoted complete regression of high-grade lesion, but adjuvanted SLPs could not exert effective therapeutic effects in most patients with established cervical cancers [27]. TA-CIN (HPV16 L2E6E7 fusion protein) is another vaccine that presented promising results in pre-clinical studies. Immunization with this antigen conferred protection to tumor outgrowth with one dose of TA-CIN plus adjuvant (Novasome) administered 4 h after the challenge with TC-1 cells [28]. However in clinical trials this vaccine was weakly immunogenic and induced low CTL responses [29–31].

The peptide based vaccines have emerged as promising approaches for the development of therapeutic HPV vaccine as they are considered to be safe, easy to produce and specific [32]. Nonetheless, studies have reported that the systemic administration of vaccines consisting of short peptides (typically nine amino acid residues) may result in T cell tolerance due to the probable binding of the peptide to non-professional APC. Also the short peptides are susceptible to rapid degradation by tissue and serum peptidases. To overcome this limitation, studies have suggested the use of long synthetic peptides that can be processed by professional APC, hereby promoting durable adaptive immune responses against multiple antigens [33]. As aforementioned, long peptide vaccines consisting of E6 and E7 epitopes have proven to be highly immunogenic when provided together with adjuvant or imune-stimulating molecule [15,26,28,29].

Our approach to overcome the limited immunogenicity of the HPV antigens consisted of a recombinant protein containing overlapping MHC I and II epitopes of E6 and E7. In this strategy, epitopes are linked together by a short amino acid motif (AAY) which are a proper substrate for proteasome mediated cleavage or binding to the transporter associated with antigen processing (TAP) [34]. After the cleavage behind the AAY motifs, the C-terminal regions of the epitopes are suitable sites for binding to TAP transporter or other chaperons [35]. Moreover, the length of the E6E7 protein is ideal to force its processing and presentation by DCs, resulting in a strong induction of T cell immune response [36]. In addition to aforementioned characteristics, our protein covers a range of epitopes from all of the possible restriction molecules expressed by HLA A, HLA B and HLA C. Thus, this vaccine has the potential to induce broad immune responses, at both individual and population levels.

In fact, the multi-epitope E6E7 protein has proven to be highly immunogenic in mice without the enhancement of any adjuvant, as three doses of vaccination was sufficient to promote full prophylactic and therapeutic anti-tumor protection in mice. The inclusion of epitope restricted to the H-2Db allele (E7_49-57_) along to the HLA- restricted epitopes of E6 and E7 seems to have collaborated substantially for the efficient protection of the E6E7 vaccine against the TC 1 cells in mice. Indeed, this E7 immunodominant epitope (RAHYNIVTF) has been previously used in several therapeutic vaccines that were tested in C57BL/6 mice, resulting in excellent immune protection against tumor which expresses HPV-16 E7 protein [37].

Although the immunodominant epitopes of E7 are likely to be critical for the immune response conferred by the E6E7 vaccine in mice, the selected epitopes of E6 have also contributed to the vaccine efficacy, as we can observe high levels of IFN-γ production by PBMCs, derived from E6E7 immunized animals, stimulated with E6_49-57_ peptide. Indeed, following the same prophylactic protocol here described, a vaccine consisting of only E6 epitopes was capable to protect 20% of the mice against tumor growth (data not shown), being a indicative of the significance of the present selected E6 and E7 epitopes as a whole to induce the activation of tumor-specific T cells.

As observed in the prophylactic assay, the vaccination of CD4^+^ T-cells knockout mice did not reduce the anti-tumor effects. Despite CD4^+^ T-cells of being crucial for the maintenance of the immune response through secretion of cytokines, such as IL-2 and IFN-γ, and for playing an important role in anti-tumor effect against genital warts and cervical cancer [38], studies have shown that the protection against immortalized cells expressing E6 and E7 is more dependent on CD8^+^ T-cells than CD4^+^ T-cells response [39]. Also, a DNA vaccine encoding E7 fused to heat shock protein (HSP), generated an E7-specific CD8^+^ T-cell responses in the absence of CD4^+^ T-cells [40]. Similarly, in our study the antitumor response generated by E6E7 vaccination required participation of CD8^+^ T-cells, but not CD4^+^ T-cells.

Despite these encouraging results in mice, the multi-epitope E6E7 vaccine will probably require the reinforcement of adjuvants to be effective in clinical trials. However, due to the high immunogenicity of the E6E7 antigen, the effectiveness of an adjuvant or an immune stimulating fusion molecule could not be evident in this current study. Actually, as an attempt to enhance the Class I MHC antigen presentation, we have produced a fusion protein composed of E6E7 and ubiquitin. In preliminary studies, this strategy seemed to increase the activation of E6/E7-specific CD8+ T-cells *in vitro* and resulted in a similar antitumor effect when compared to E6E7 (data not shown).

In an effort to circumvent the poor immunogenicity of peptide or protein-based vaccines, much of the research in this area has explored strategies employing adjuvants or fusion protein [41]. The combined administration of synthetic long peptides with Montanide and CpG resulted in a powerful protective immune response in mice [26]. Another previously study, the activation of dendritic cells and induction of T cell responses by HPV 16 L1/E7 chimeric virus-like particles were enhanced by CpG-ODN or sorbitol [42]. Several other fusing strategies (heat shock proteins, calreticulin, herpesvirus glycoprotein D) have been effective in increasing CTL response and have generated antitumor immunity of the antigens in animals [5,43–46].

In this current study, we demonstrated that immunization of mice with the E6E7 protein containing CTL and T helper epitopes of E6 and E7 can promote high activation of specific T-cells and can efficiently inhibit the tumor growth in a prophylactic and therapeutic context. Hereby, the immunogenicity of this E6E7 antigen can be intensely explored to reach the idealized vaccine against cervical tumor cells.

## Supporting Information

S1 Fig Peptides identified by mass spectrometry. The aminoacid sequences of the E6E7 protein. E6 (blue) and E7 (green) peptides are distinguished by color. Peptides identified by mass spectrometry are underlined.
(TIF)

S1 Material and Methods Mass spectrometry analyses. (DOCX)

S1 Table Peptides identified by mass spectrometry. (TIF)
