# Supplementary figures and images for: Design, Immune Responses and Anti-Tumor Potential of an HPV16 E6E7 Multi-Epitope Vaccine
Source: PLoS One. 2015 Sep 21;10(9):e0138686. doi: 10.1371/journal.pone.0138686 (PMC4577214; doi:10.1371/journal.pone.0138686)

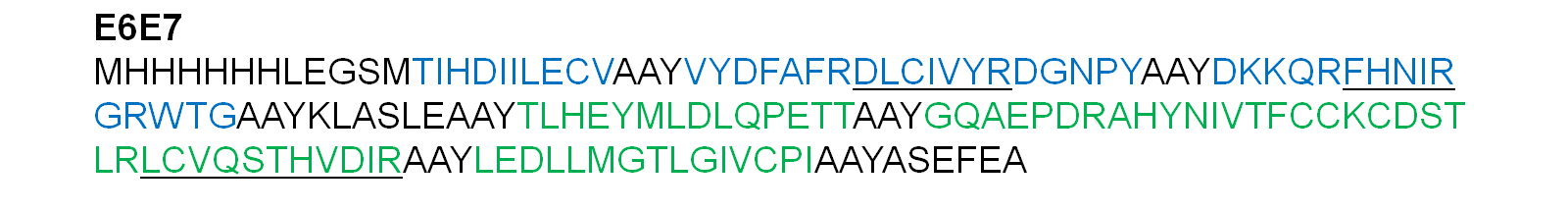

Supplement: S1 Fig — The aminoacid sequences of the E6E7 protein. E6 (blue) and E7 (green) peptides are distinguished by color. Peptides identified by mass spectrometry are underlined. (TIF) [file pone.0138686.s001.tif]

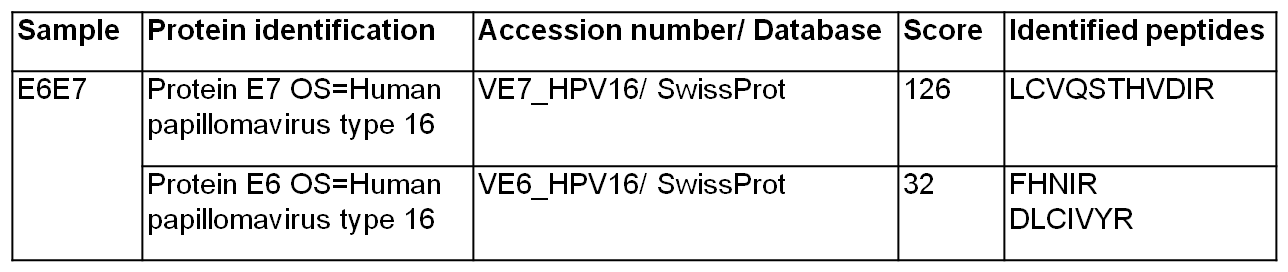

Supplement: S1 Table — (TIF) [file pone.0138686.s003.TIF]
