# Supplementary material for: Design, Immune Responses and Anti-Tumor Potential of an HPV16 E6E7 Multi-Epitope Vaccine
Source: PLoS One. 2015 Sep 21;10(9):e0138686. doi: 10.1371/journal.pone.0138686 (PMC4577214; doi:10.1371/journal.pone.0138686)
Supplement: S1 Material and Methods — (DOCX) [file pone.0138686.s002.docx]

**S1_ Material and Methods. Mass spectrometry analyses.** Protein band was excised from SDS-PAGE gel and in-gel trypsin digestion was performed according to literature (1). After digestion procedures, the peptide mixture was desalted using Zip-Tip® C18 (Millipore) according to manufacturer´s instructions. An aliquot (4.5 µL) of resulting peptide mixture was separated by nanoAcquity UPLC® BEH130 C18 column (75 µm i.d. x 100 mm) (Waters, Milford, MA, USA) on a UPLC-ESI-Q-TOF system (Waters) at a flow rate of 600 nL/min; or eventually on a nanoLC (EksigentTechnologies) coupled to LTQ XL™ Linear Ion trap (Thermo Scientific). In both instruments a gradient of 3-45% of solvent B (0.1% formic acid in acetonitrile), 45-80% B in 2.5 min, hold at 80% B for 1 min, then back to 97% of solvent A (0.1% formic acid in deionized water) in 1.5 min were used. The MS instruments were operated in data dependent mode, in which one full MS scan was acquired in the m/z range of 200-2000 Da followed by MS/MS acquisition using collision induced dissociation. The resulting fragment spectra were searched using free MASCOT search engine (Matrix Science) using viruses as taxonomy against NCBInr (1309992 sequences) and SwissProt (16459 sequences), with a parent and fragment tolerance of 1.2 and 0.6 Da, respectively. Iodoacetamide derivative of cysteine and oxidation of methionine were specified in MASCOT as variable modifications.

**Reference**

1. Hanna SL, Sherman NE, Kinter MT, Goldberg JB. Comparison of proteins expressed by Pseudomonas aeruginosa strains representing initial and chronic isolates from a cystic fibrosis patient: an analysis by 2-D gel electrophoresis and capillary column liquid chromatography-tandem mass spectrometry. Microbiology 2000;146 ( Pt 10):2495-508.
